# More schooling is associated with lower hemoglobin A1c at the high-risk tail of the distribution: an unconditional quantile regression analysis

**DOI:** 10.1186/s12889-025-23063-x

**Published:** 2025-06-03

**Authors:** Jillian Hebert, Amanda M. Irish, Aayush Khadka, Abigail Arons, Alicia R. Riley, Elbert S. Huang, Anusha M. Vable

**Affiliations:** 1 Department of Family and Community Medicine, University of California, 2540 23rd St, 94110 San Francisco, CA USA; 2 Departments of Internal Medicine and Pediatrics, University of California, San Francisco, San Francisco, CA USA; 3 Department of Sociology, University of California, Santa Cruz, Santa Cruz, CA USA; 4 Departments of Medicine and Public Health Sciences, University of Chicago, Chicago, IL USA; 5 The Philip R. Lee Institute of Health Policy Studies, University of California, San Francisco, San Francisco, CA USA

**Keywords:** Unconditional quantile regression, Distributional effects, Effect heterogeneity, Diabetes, US Health and Retirement Study (HRS)

## Abstract

**Background:**

Risk of diabetes increases exponentially with higher levels of glycosylated hemoglobin (HbA1c). Education is inversely associated with average HbA1c, however, differential associations between education and HbA1c across the HbA1c distribution have not been evaluated.

**Methods:**

Health and Retirement Study data (*N* = 21,732) was used to evaluate the association between education (linear terms among those with < 12 years and ≥ 12 years of education) and first recorded HbA1c (2003–2016) at the mean using linear regression, and at the 1st-99th quantiles of the marginal outcome distribution using unconditional quantile regressions, controlling for birth year, race and ethnicity, gender, birthplace, parental education, and year of HbA1c measurement.

**Results:**

Mean HbA1c was 5.9%; 16.6% of participants had HbA1c above the diabetes diagnostic threshold of 6.5%. For those with fewer than 12 years of schooling, there was no association between education and HbA1c at the mean or across the quantiles. For those with 12 or more years of schooling, an additional year of education was negatively associated with mean HbA1c (β_OLS_=-0.02, 95% confidence interval (CI) -0.03,-0.02); a one-year increase in mean education was associated with lower HbA1c across the distribution, but the magnitude was larger at higher quantiles (β_q50_=-0.02, 95%CI -0.02,-0.01; β_q90_=-0.06, 95%CI -0.09,-0.04).

**Conclusions:**

Educational attainment is inversely associated with HbA1c among those with 12 or more years of schooling, with larger point estimates for those in the high-risk tail of the HbA1c distribution.

**Supplementary Information:**

The online version contains supplementary material available at 10.1186/s12889-025-23063-x.

## Introduction

Prior work, both in the US and globally, reveals a strong inverse relationship between educational attainment and average glycosylated hemoglobin (HbA1c), an index of glucose regulation over the past 2–3 months, such that those with less education are at higher risk of diabetes and diabetes-related complications [1, 2, 3, 4, 5, 6]. Among people with diabetes, glycemic control is essential for reducing complications, such as kidney failure, cardiovascular events, and all-cause mortality; [7, 8, 9, 10, 11] in parallel, staying within normal ranges of HbA1c is important to prevent diabetes onset and related complications. Risk of diabetes complications increase exponentially with HbA1c, such that a percentage-point increase at a higher level of HbA1c (e.g. 6–7%) confers a much greater risk for diabetes-related complications compared to a percentage-point increase at lower HbA1c levels (e.g. 4–5%). Therefore, exposures and interventions that have larger impacts for those with higher HbA1c may be important for preventing diabetes-related complications.

Education, a main component of socioeconomic status, is consistently associated with better health over the lifecourse [12, 13]. Increased educational attainment may impact HbA1c through several pathways, including longer life expectancy, increased income, better access to healthcare, and more health-promoting behaviors (e.g., increased physical activity). [1, 4, 14, 15, 16] While evidence suggests a strong protective association between educational attainment and mean HbA1c, no study evaluates the association across the entire HbA1c distribution. Evaluating if the relationship is constant across the HbA1c distribution is important, given the exponential relationship between HbA1c and diabetes onset and related complications. Interventions that specifically impact high levels of HbA1c are of interest given their potential to reduce diabetes onset and related complications. We hypothesize education will have larger associations among those with higher HbA1c as participants belonging to more structurally minoritized subgroups (e.g., minoritized due to race or poverty status) will be over-represented at the high-risk end of the HbA1c distribution (i.e., higher quantiles of the HbA1c distribution), [17, 18, 19] and these same groups also seem to benefit more from education. [20, 21, 22].

We add to existing literature by evaluating the relationship between education and HbA1c across the HbA1c distribution through a novel application of quantile regression, a modeling technique that evaluates the exposure-outcome relationship across the outcome distribution. Quantile regressions can identify if educational attainment has a heterogeneous effect across the HbA1c distribution, which allows for discovery of whether, and which parts of, the HbA1c’s distribution are differentially impacted by educational attainment. In this way, our application of quantile regression to the education-HbA1c relationship has the potential to deepen our understanding of educational inequities in diabetes by uncovering details hidden by linear regression.

This paper empirically evaluates the relationship between educational attainment and late-life HbA1c using linear regression and unconditional quantile regressions (UQR) for US Health and Retirement Study participants ages 50 and older, examining whether the relationship between education and HbA1c varies across the HbA1c distribution. We stratify education at 12 years, where 12 years of schooling typically confers a high school diploma, since prior literature finds evidence for divergent health trajectories of adults with less than a high school education from those with high school or more. [23].

## Methods

### Data and analytic sample

Data came from the US Health and Retirement Study (HRS), a national longitudinal sample of non-institutionalized adults 50 years and older, and their spouses of any age, that began in 1992. [24] New cohorts of participants have been added every six years after 1998 to maintain a steady state population, and participants are surveyed biennially. A diabetes substudy collected HbA1c for a subset of HRS participants in 2003; in addition, HbA1c and other biomarker data was collected in 2006 for a randomly selected half of the sample, and in 2008 for the other half; biomarker data were subsequently collected every four years.

The eligible sample included all HRS participants with at least one HbA1c measurement between 2003 and 2006 when they were 50 years or older (*N* = 21,840). Individuals were excluded for missing exposure (*N* = 89) and covariate data (*N* = 18); one participant was removed due to their HbA1c measurement being recorded prior to their first HRS interview, resulting in an analytic sample of 21,732 participants (99% of eligible).

### Exposure

Our exposure, educational attainment, was created using self-reported total years of schooling. Education in HRS ranges from 0 to 17 years of schooling, where 17 years includes those with 17 or more years of education (17 or more years: *N* = 2,327). Due to data sparseness of participants with fewer than 5 years of education, we coded those with fewer than 5 years of education to 5 years to reduce the impact these outliers may have on estimates (*N* = 776). To assess possible heterogeneities between participants with different types of credentials, and since educational policies tend to target specific levels of education (e.g. compulsory schooling laws and child labor laws targeted K-12 education, while other policies only addressed college education), education was stratified into two levels: fewer than 12 years of education (*N* = 4,801; 22%) and 12 or more years of education (*N* = 16,931; 78%), where completing 12 years of education typically corresponds to earning a high school diploma. Education was modeled linearly within these two educational strata.

### Outcome

Our outcome was the participant’s first recorded HbA1c value (2003–2016) measured at or after age 50. HbA1c is glycosylated hemoglobin and reflects blood glucose over the prior 2–3 months; HbA1c values between 5.71% and 6.49% are consistent with pre-diabetes and values greater than 6.5% are consistent with diabetes [25, 26, 27]. HbA1c was measured using automated ion-exchange high-performance liquid chromatography that recorded the percentage of glycosylated hemoglobin in dried blood spot samples [24].

### Covariates

All models were adjusted for sex (female; male), race (Non-Hispanic White; Non-Hispanic Black; Latinx/Hispanic; other), birthplace (non-Southern US; Southern US; foreign), indicator for birth year (1905–1966), indicator for year of HbA1c measurement (2003–2016), mother’s education (5–17(+) years, linear), father’s education (5–17(+) years, linear), as well as missing indicators for mother’s education and father’s education. Sex was included as an indicator of the socially stratifying effects of gender [28], race as an indicator of the socially stratifying effects of systemic racism [29–30], and parent’s education as a proxy for childhood socioeconomic status. Chi-square tests were used to evaluate if there were significant differences in categorical covariates by education level (e.g., less than 12 years of education versus 12 or more years of education). See supplemental Table S1 for additional details on covariates.

Race was categorized to include an “other” category in all models for precision, but results were not reported for this group due to the heterogeneous composition and consequent lack of interpretability of estimates.

Birthplace was classified by location within the US (i.e., non-Southern vs. Southern) because studies have found increased risk for adverse later-life health outcomes for those born in the Southern US [31, 32, 33, 34]. A subset of participants (*N* = 414, 2%) were known to be born in the US, but were missing information on the region of birth; participants where the region of birth was unknown were assumed to be born in the non-Southern US.

Birth year was modeled as an indicator variable to capture differences by individual year. Due to a small number of participants falling in the tail ends of the birth year range, values were recoded to facilitate model convergence: those born before 1917 (*N* = 240, 1%) were recoded as 1917; those born in 1966 (*N* = 52, 0.2%) were recoded as 1965.

Parental education variables (5–17(+) years) were used as a proxy for family socioeconomic status (SES) and modeled continuously. However, HRS participants in the Asset and Health Dynamics Among the Oldest Old (AHEAD) cohort (born 1900–1923) recorded parent’s education as a dichotomized measure (less than 8 years of education, 8 or more years of education) rather than continuous. Dichotomized measures of parent’s education were replaced with continuous values from a previously validated imputation method using measures of childhood socioeconomic status. [35] Additional missingness in parental education was imputed using the sample mean (mother’s education: N_Missing_ = 2,101 (10%), sample mean = 10 (SD 3.9); father’s education: N_Missing_ = 3,644 (17%), sample mean = 10 (SD 4.2)) and a missing indicator was added for proper model adjustment. This allowed for retention of participants with missing parental education where missingness is informative (e.g., if the parent was not in the household). [36].

### Statistical analysis

We used linear regressions and unconditional quantile regressions (UQR) to model the relationship between education and HbA1c [37, 38, 39]. UQR evaluates changes in quantiles of the outcome’s unconditional distribution for a one-unit change in the mean of the exposure. We estimated parameters of the linear regression model using ordinary least squares (OLS). We fit UQR models at each unit quantile between the 1st-99th quantiles of the unconditional HbA1c distribution. We used bootstrapping (500 repetitions) to estimate 95% confidence intervals (CIs) for parameters of the linear regression and UQR models; education was modeled as a linear term within both education strata and all models were adjusted for the covariates specified in the preceding section.

To visualize the change in the sample distribution of HbA1c implied by UQR results, we created plots to show the counterfactual HbA1c distribution for a one-year increase in the sample’s mean education. First, we binned the factual, or observed, data into quantiles (1st-99th). We then added the UQR estimation of the association between education and HbA1c to the observed data by quantile, creating a potential counterfactual distribution. Finally, we plotted the observed and counterfactual distributions. Further details about constructing these datasets and plots are provided elsewhere. [39].

### Sensitivity analysis

We conducted three additional analyses to see if results were robust to different analytic decisions. First, to determine if results were sensitive to how the exposure was operationalized (i.e., coded for analysis), we re-coded education as a three category variable (< 12 years, 12–15 years, and 16 or more years of education) rather than two. Second, given that the HRS collects data from numerous birth cohorts (from 1905 to 1966) and given that educational attainment has tended to increase over time, we conducted analyses stratified by HRS entry cohort (which are defined by the HRS using participant’s birth year), to test the sensitivity of the association to secular trends in education. Finally, while medication could be an important contributing factor in the education-HbA1c relationship, medication usage is downstream from education and is therefore a potential mediator of the education-HbA1c relationship. Adjusting for mediators can bias estimates, [40] so the main analyses did not include adjustment for medication.

### Software and code

All data cleaning and analysis was performed in R. [41] We used the *dineq* package for fitting UQRs. All code was reviewed by the second author as is recommended practice [42] and can be found on GitHub.

## Results

Participants included in the analysis (Table 1) had an average of 13 years of education, were predominantly women (57%), White (65%), and born in the non-Southern US (53%). Most participants self-reported having 12 or more years of education (78%) and median HbA1c (interquartile range - IQR) in the overall sample was 5.7% (5.3-6.2%). Compared to participants with 12 or more years of education (henceforth 12 + years), those with fewer than 12 years of education (henceforth < 12 years) were older (mean age: 67 vs. 65), had higher proportions of Black and Latinx participants (23% vs. 17% Black; 31% vs. 8% Latinx), had a higher proportion of Southern US birthplace (42% vs. 31%), and had lower parental education (mean mother’s education: 8 vs. 11; mean father’s education: 8 vs. 10) with higher rates of missingness (20% vs. 7% missing mother's education; 30% vs. 13% missing father's education). Median HbA1c (IQR) for < 12 years was 5.8% (5.5-6.4%) and 5.6% (5.3-6.1%) for 12 + years. Chi-square tests found significant differences in race and ethnicity, birthplace, proportion missing parent’s education, and proportion of the sample with HbA1c levels that are consistent with being pre-diebetic or having diabetes.

**Table 1:** Distribution of covariates in the analytic sample

	Overall *N* = 21,732	Fewer than 12 Years of Education *N* = 4,801; 22%	12 or More Years of Education *N* = 16,931; 78%	Chi-Squared *p*-value
**Educational Attainment (Years)**	12.7 (3.0)	8.5 (2.1)	13.9 (1.9)	
**Age (Years)**	65.1 (10.6)	67.2 (11.0)	64.5 (10.4)	
**Average Birth Year**	1944 (12.2)	1942 (12.5)	1944 (12.0)	
**Proportion Female**	57%	57%	57%	0.7295
**Proportion in each Race and Ethnicity**				< 0.0001
** Non-Hispanic White**	65%	43%	71%	
** Non-Hispanic Black**	18%	23%	17%	
** Latinx/Hispanic**	13%	31%	8%	
** Other**	4%	3%	4%	
**Proportion in each Birthplace**				< 0.0001
** Non-Southern US**	53%	32%	59%	
** Southern US**	34%	42%	31%	
** Foreign**	13%	26%	10%	
**Average Mother’s Education**	10.1 (3.1)	8.1 (2.7)	10.7 (2.9)	
**Proportion Missing Mother’s Education**	10%	20%	7%	< 0.0001
**Average Father’s Education**	9.9 (3.2)	8.1 (2.6)	10.4 (3.2)	
**Proportion Missing Father’s Education**	17%	30%	13%	< 0.0001
**HbA1c Measurement Year**				
** 2003**	6%	8%	5%	
** 2006**	27%	26%	27%	
** 2008**	27%	28%	26%	
** 2010**	14%	14%	14%	
** 2012**	13%	12%	14%	
** 2014**	5%	5%	5%	
** 2016**	8%	7%	9%	
**Median HbA1c (IQR)**	5.7% (5.3-6.2%)	5.8% (5.5-6.4%)	5.6% (5.3-6.1%)	
**Proportion Pre-Diabetic** **(HbA1c between 5.71-6.49%)**	30%	34%	29%	< 0.0001
**Proportion Diabetic (HbA1c ≥ 6.5%)**	17%	23%	15%	< 0.0001
**Medication (Yes)**	19%	28%	17%	< 0.0001

Note: Reported average mother and father’s education includes participants with missing values that were replaced with the sample mean (mean of 10 years for both mother’s and father’s education). Chi-square tests are used to compare significant differences in categorical variables by education level.

Abbreviations: IQR = interquartile range

Table 1.

**Fig. 1:**
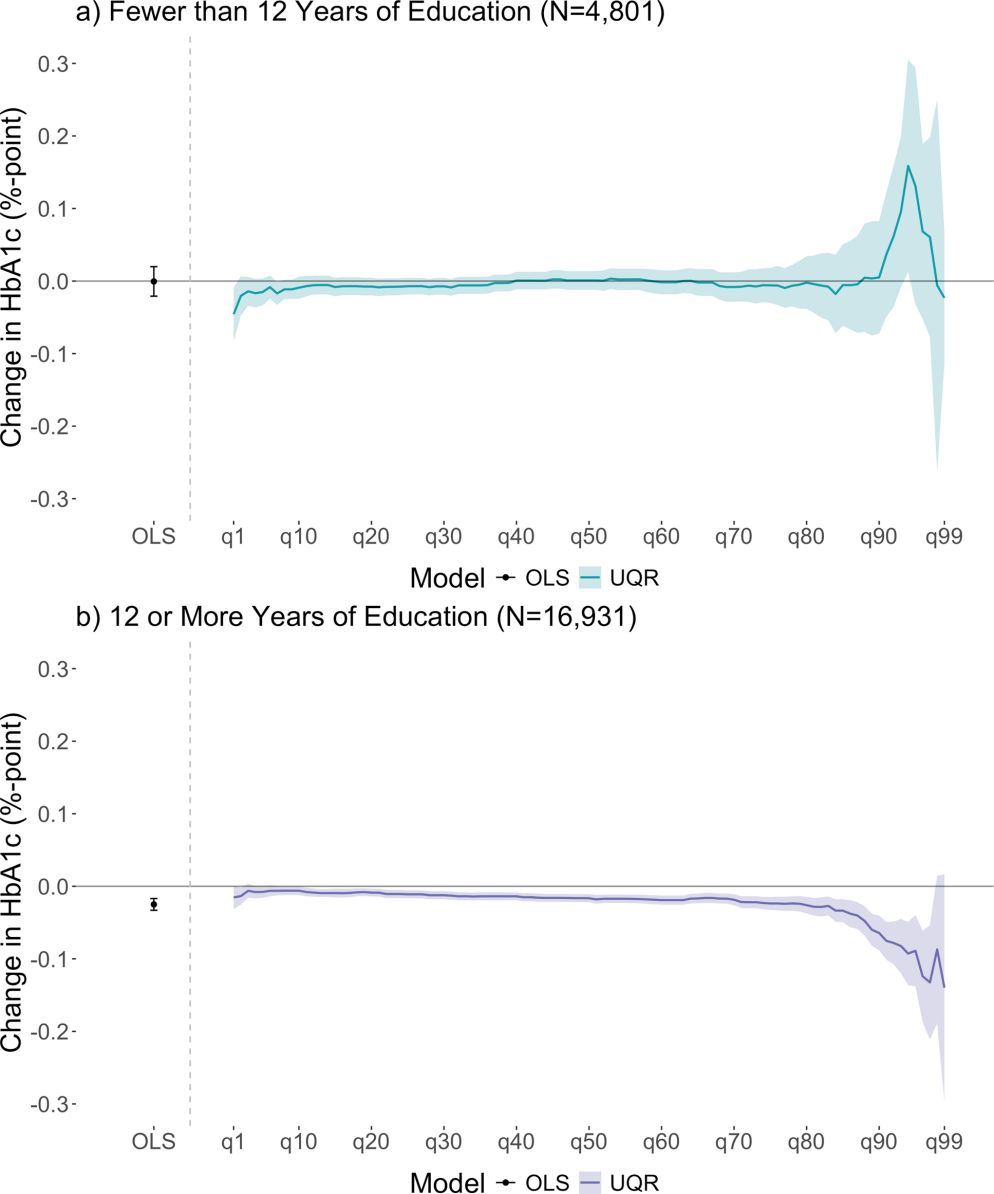
Comparing results from linear regressions estimated using OLS with unconditional quantile regressions by dichotomized education. Note: Panel a) presents linear regression and UQR estimates with associated 95% confidence intervals for participants with fewer than 12 years of education. Panel b) presents linear regression and UQR estimates with associated 95% confidence intervals for participants with 12 or more years of education. OLS stands for Ordinary Least Squares. q10 = 10th quantile, q20 = 20th quantile, and so forth. The 90th quantile in the HbA1c distribution for those with less than 12 years of education was 7.7%. The 90th quantile in the HbA1c distribution for those with 12 or more years of education was 6.9%. The point to the left of the vertical dashed gray line in each panel represents the point estimate from OLS with 95% confidence intervals. The solid line to the right of the vertical dashed gray line in each panel represents point estimates from unconditional quantile regressions and shaded areas represent the 95% confidence intervals fit at each unit quantile between the 1st-99th quantiles of the HbA1c distribution. All models were adjusted for covariates and 95% confidence intervals were estimated using bootstrapping (500 resamples). Confidence intervals at the tails of the distribution are larger due to sparse data (i.e., fewer participants falling in those quantiles)

Figure 1 displays linear regression and UQR results for both education strata (< 12 years; 12 + years). Linear regression results indicate that each additional year of education was not associated with average HbA1c for participants with < 12 years (Fig. 1, panel a: -0.00, 95% CI: -0.02, 0.02). UQR results for the 1st-99th quantiles suggest that a one-year increase in mean education was not associated with HbA1c across most quantiles, but may have been associated with higher HbA1c at higher quantiles (91-97th quantiles), although the confidence intervals here were wide (Fig. 1, panel a: β_q5_ = -0.02%; 95% CI: -0.03, 0.03, β_q50_ = 0.00; 95% CI: -0.01, 0.01, β_q95_ = 0.13%; 95% CI: -0.03, 0.29).

For participants with 12 + years, linear regression results indicate that each additional year of education was associated with lower average HbA1c (Fig. 1, panel b: -0.02, 95% CI: -0.03, -0.02). UQR results suggest that a one-year increase in mean education was associated with lower HbA1c across almost all quantiles with a larger magnitude at higher quantiles (90-99th quantile) (Fig. 1, panel b; β_q5_ = -0.01%; 95% CI: -0.02, -0.00, β_q50_ = -0.02%; 95% CI: -0.02, -0.01, β_q95_ = -0.09%; 95% CI: -0.14, -0.04).

**Fig. 2:**
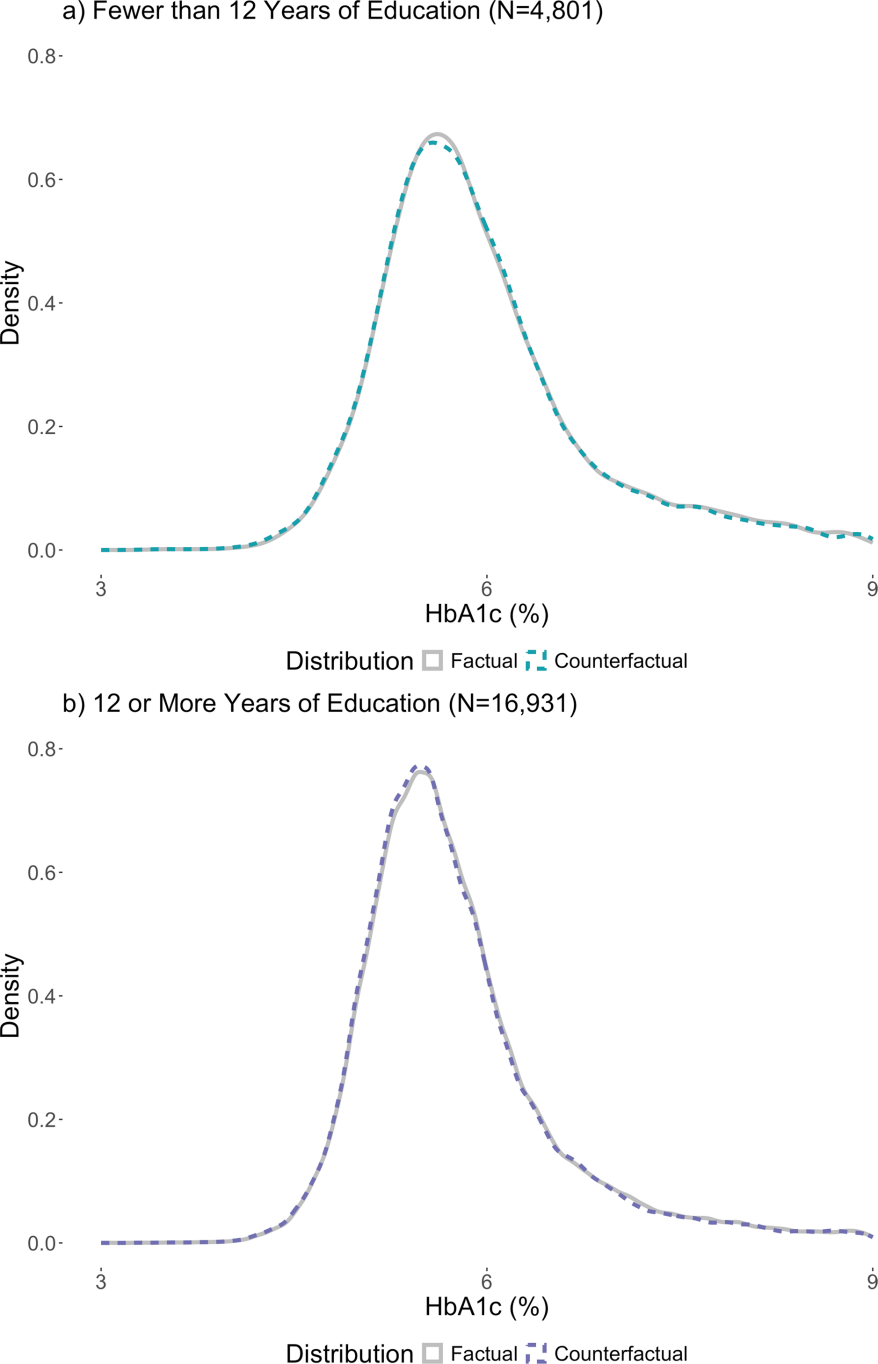
Peaks of the c ounterfactual HbA1c distributions based on unconditional quantile regression estimates by dichotomized education. Note: Panel a) presents the factual and counterfactual distribution of HbA1c for participants with fewer than 12 years of education. Panel b) presents the factual and counterfactual distribution of HbA1c for participants with 12 or more years of education. These graphics cut off the tails of the entire distribution to focus on the peak of the HbA1c distribution. The full HbA1c range for fewer than 12 years of education is 3.4–17.0% and the full HbA1c range for 12 or more years of education is 3.0–18.2%. Counterfactual HbA1c distributions were created using quantile estimates from UQR models. Counterfactual distributions were created to help visualize the reshaping of the HbA1c distribution for a one-year increase in the average educational attainment in the sample

Figure 2 illustrates the factual, or observed, distribution of HbA1c in the sample and the predicted reshaping (i.e., counterfactual) of the HbA1c distribution based on the UQR estimates. Figure 2 helps to visualize the potential impact of increased education on the HbA1c distribution (i.e., the counterfactual distribution). Consistent with the magnitude of the UQR estimates in Fig. 1, changes in the counterfactual HbA1c distribution are small and therefore difficult to discern; to aid in visualization, Fig. 2 zooms in on the peak of the HbA1c factual and counterfactual distributions. A figure including the full HbA1c distribution is included in the appendix (eFigure 1). For those with < 12 years, the density at the peak of the counterfactual distribution is slightly lower than the density at the peak of the factual distribution, suggesting a small rightward shift away from the densest point of the distribution, towards higher HbA1c values. For 12 + years, the density at the peak of the counterfactual distribution is slightly higher than the density at the peak of the factual distribution, suggesting a small leftward shift in the direction of the densest part of the distribution, towards lower HbA1c values.

### Sensitivity analyses

Results were attenuated but substantively similar and conclusions were unchanged when stratifying into 3 education strata (< 12 years of education, 12–15 years of education, 16 + years of education) (eFigure 2). Results for 12–15 years of education were most similar to results for the 12 + years of education in the main analysis; results for 16 + years of education were null across most quantiles but were associated with lower HbA1c at higher quantiles (90-98th quantiles).

When evaluating differences by entry cohorts, most results included the null, but estimates were largely in the same direction (eFigures 3–9). The War Babies (birth years: 1942–1947) and Mid Baby Boomer (birth years: 1954–1959) cohorts’ results were most dissimilar to the main results.

Results and conclusions were unchanged after additional adjustments for medication (eFigure 10).

## Discussion

In the HRS sample of older US adults, we found that the relationship between educational attainment and HbA1c varies by education level and is heterogeneous across the HbA1c distribution. Overall, associations between education and HbA1c seem to be strongest at the high-risk tail of the HbA1c distribution (i.e., participants with the highest HbA1c levels; 90-99th quantiles). That is, participants with the highest HbA1c levels who are most at risk of developing diabetes or experiencing diabetes-related complications such as cardiovascular events and all-cause mortality had the strongest association with educational attainment. We also found that these associations varied by total years of schooling: among those with fewer than 12 years of education, a one-year increase in mean education was not associated with HbA1c for participants with low-to-average HbA1c levels, however, it was weakly associated with higher HbA1c for participants with high HbA1c levels. Conversely, among those with 12 or more years of education, a one-year increase in mean education was associated with lower HbA1c across the distribution, with larger magnitudes in the association at the high-risk tail of the distribution; more education was associated with a leftward shift in the HbA1c distribution, suggesting lower risk of diabetes and diabetes-related complications in the sample population. While the associations appear minimal, our work adds to existing literature by demonstrating a method of evaluating heterogeneous relationships and identifying that the relationship between education and HbA1c differs by education level.

There were two analytic decisions that allowed us to identify these novel findings. First, we stratified our exposure at 12 years of education. This stratification was informed by prior research highlighting the divergent health trajectories of adults with less than a high school education from those with high school or more. [23] Second, we used UQRs to evaluate the relationship across the entire HbA1c distribution, as opposed to evaluating the relationship at the mean, where one assumes the change at the mean is constant across the entire HbA1c distribution. Among older adults with more than 12 years of education, estimates at the mean were associated with lower HbA1c, as were estimates for most of the quantiles in the HbA1c distribution; at the highest quantiles of the HbA1c distribution, magnitudes of the estimates were larger, suggesting that there may be a larger association for those with higher levels of HbA1c. This discovery additionally exemplifies that the mean association was not constant across the entire HbA1c distribution.

Our finding that increased educational attainment was associated with lower HbA1c among those with 12 or more years of education is consistent with prior literature showing that lower educational attainment is associated with increased risk of diabetes and more broadly that detrimental social factors strongly affect risk of diabetes [1, 2, 3, 4, 5, 6, 7, 8, 9, 10, 11, 12, 13, 14, 15, 16, 17]. Our use of quantile regressions revealed a larger magnitude of the education-HbA1c association at the high-risk tail of the HbA1c distribution, where individuals are more likely to have diabetes. We hypothesize two potential mechanisms for this: first, the impact of structural factors (race, poverty, historic systems of marginalization and exclusion) on educational attainment and HbA1c. Evidence shows that education has a larger impact among low childhood socioeconomic status or racial and ethnic minoritized groups when compared to structurally advantaged groups (e.g., White) [43, 44, 45, 46, 22]. Second, education impacts skill-level, which in turn impacts the type of job one attains and one's income. Therefore, individuals with diabetes who have higher levels of education likely also have greater access to resources, such as money, health care and medications, or social and behavioral resources that support lifestyle changes to better manage glucose levels [47, 20, 21].

Our findings about the relationship between HbA1c and education are relevant for both clinicians and public health policymakers. At the clinical level, this association underscores the role of social determinants of health screening as a part of diabetes risk assessment, and future research should explore the added benefit of more precise screening for education in terms of number of years rather than the more commonly used broad categories of educational attainment. Such screening and subsequent linkage to social services has the potential to support clinicians in better identifying and supporting patients to lower their risk of diabetes (e.g., pursuing earlier or more intensive preventive intervention or more aggressive glycemic control once diagnosed with diabetes). Additional studies could explore the effectiveness of interventions for these patients that seek to mitigate the detrimental effect of less education on HbA1c, such as disease education programs, navigational supports, and pharmacy management tools. Finally, our results raise the question of whether increasing educational attainment, even by one year, for populations would be an effective intervention to reduce future glucose levels. Future secondary data analyses and intervention studies can further elucidate whether individual-level or population-level interventions that increase post-secondary educational attainment may be an effective strategy for lowering diabetes risk.

It is important to contextualize why results for those with fewer than 12 years of education are different from those with 12 or more years of education, especially because the results for those with fewer than 12 years of education are contrary to our hypothesis and prior literature. It could be that those with fewer than 12 years of education are a different, more structurally minoritized group than those with more education, resulting in different relationships between education and HbA1c; we found significant differences in race and ethnicity, birthplace, proportion missing parent’s education, and proportion of the sample with HbA1c levels that are consistent with being pre-diebetic or having diabetes. Our sample of those with fewer than 12 years of education were more likely to be people of color, born outside of the US or in the Southern US, and their parents had less education or more missing data on education – all potential indicators of increased marginalization. Results could also be due to standard variability in the unconditional quantile regression estimates at the tails of the distribution, where the density is low, and the variance of the recentered influence function (RIF) is larger [37].

An important strength of this analysis is the additional information provided by the UQR modeling technique. By looking at the exposure-outcome relationship across the entire outcome distribution, estimates can identify heterogeneities in the education and HbA1c relationship. OLS results only suggest lower HbA1c levels given an additional year of education for participants with 12 or more years of education. UQR results further suggest that the magnitude of change in HbA1c is larger for those at the high-risk tail of the distribution. Comparing the OLS and UQR results highlights the limitation of mean models in capturing an exposure’s relationship with the outcome distribution, and the necessity for evaluating the relationship across the entire outcome distribution, especially when the risk could be non-linear.

There are limitations in these analyses that should be acknowledged. As with any observational study, residual confounding is a potential problem. We did not have information on whether participants had type 1 or type 2 diabetes; inability to differentiate if participants had type 1 or 2 diabetes was a concern given that they are two distinct diseases with unique risk factors and treatments. Additionally, we evaluated educational quantity, and assumed quality was comparable across respondents, potentially resulting in residual confounding in the relationship between educational attainment and HbA1c. We hypothesize that the education-HbA1c relationship could differ by race and ethnicity and sex, due to socially stratifying effects of gender and systemic racism; understanding if there are differential returns to education by sociodemographic subgroup is an important area for future study. Results from HRS underscores that these analyses should be replicated in other data sources to determine if these results are robust to variations in time, place, and population; evaluating if these relationships are causal is also warranted.

Our results suggest the relationship between education and HbA1c is heterogeneous, varying both by education level and across the HbA1c distribution, with the largest associations in the high-risk right tail of the HbA1c distribution where risk of diabetes-related complications is highest. We found a one-year increase in average education for those with 12 or more years of education was associated with lower HbA1c, with larger point estimates for those in the high-risk tail of the HbA1c distribution. Our results add to the understanding of the education-HbA1c relationship and underscore the importance of evaluating the education-HbA1c relationship across the entire outcome distribution. Our results may also suggest an avenue for intervention: policies to increase education could reduce population-level diabetes complications, such as cardiovascular events and all-cause mortality.

## Electronic supplementary material

Below is the link to the electronic supplementary material.

Supplementary Material 1

## Data Availability

The data that support the findings of this study are available from the U.S. Health and Retirement Study (HRS) but restrictions apply to the availability of these data. Both public (Cross-Wave Tracker File and RAND HRS Longitudinal File 2018) and sensitive (2003 Diabetes Study; 2006-2016 Biomarker Data) datafiles were used in analysis. The HRS is sponsored by the National Institute on Aging (grant number NIA U01AG009740) and is conducted by the University of Michigan. Access to datasets are available through the HRS website (https://hrs.isr.umich.edu). The datasets generated and analyzed during the current study are available in the “More-schooling-is-associated-with-lower-Hemoglobin-A1c-at-the-high-risk-tail-of-the-distribution” GitHub repository.

## Abbreviations

HbA1c: Hemoglobin A1c
CI: Confidence interval
