# Supplementary material for: More schooling is associated with lower hemoglobin A1c at the high-risk tail of the distribution: an unconditional quantile regression analysis
Source: BMC Public Health. 2025 Jun 3;25:2062. doi: 10.1186/s12889-025-23063-x (PMC12131358; doi:10.1186/s12889-025-23063-x)
Supplement: Supplementary file 1 — Supplementary Material 1 [file 12889_2025_23063_MOESM1_ESM.pdf]

eTable 1. Definition of covariates used in all models

**Covariate**	**Variable type**	**Definition**
Birth year	Indicator	Participant's year of birth (1917-1965); where 1905-1916 was recoded as 1917 and 1966 was recoded as 1965 due to small bin size
Gender	Categorical	1 = Female; 2 = Male
Race	Categorical	1 = Non-Hispanic White; 2 = non-Hispanic Black; 3 = Latinx / Hispanic; 4 = Other
Birthplace	Categorical	1 = Non-Southern US; 2 = Southern US; 3 = Foreign born
Mother's education	Continuous	5-17; where 5 indicates 5 or less years of education and 17 indicates 17 or more years of education
Mother's education Missing Indicator	Indicator	1 = Missing mother's education; 0 = Otherwise; Where missing mother's education was replaced with the sample mean (mean = 10)
Father's Education	Continuous	5-17; where 5 indicates 5 or less years of education and 17 indicates 17 or more years of education
Father's education Missing Indicator	Indicator	1 = Missing father's education; 0 = Otherwise; Where missing father's education was replaced with the sample mean (mean = 10)
HbA1c Measurement Year	Categorical	1 = 2003; 2 = 2006; 3 = 2008; 4 = 2010; 5 = 2012; 6 = 2014; 7 = 2016

eTable 2. Distribution of Covariates in the Sensitivity Analytic Samples

	**Overall** N = 21,732	**Less than 12 Years of Education** N = 4,801; 22%	**12-15 Years of Education** N = 12,007; 55%	**16 or More Years of Education** N = 4,924; 23%	**Chi-Squared p-value**
**Educational Attainment (Years)**	12.7 (3.0)	8.5 (2.1)	12.8 (1.0)	16.5 (0.5)	
**Age (Years)**	65.1 (10.6)	67.2 (11.0)	64.9 (10.5)	63.6 (10.1)	
**Birth Years**	1905-1966	1905-1966	1908-1966	1910-1966	
**Proportion Female**	57%	57%	60%	51%	<0.0001
**Proportion in each Race and Ethnicity**					<0.0001
Non-Hispanic White	65%	43%	69%	76%	
Non-Hispanic Black	18%	23%	18%	13%	
Latinx/ Hispanic	13%	31%	10%	6%	
Other	4%	3%	3%	5%	
**Proportion in each Birthplace**					<0.0001
Non-Southern US	53%	32%	59%	59%	

Southern US	34%	42%	32%	29%	
Foreign	13%	26%	9%	12%	
**Average Mother's Education**	10.1 (3.1)	8.1 (2.7)	10.2 (2.8)	11.8 (3.1)	
**Proportion Missing Mother's Education**	10%	20%	8%	3%	<0.0001
**Average Father's Education**	9.9 (3.2)	8.1 (2.6)	9.9 (2.9)	11.7 (3.5)	
**Proportion Missing Fathers's Education**	17%	30%	15%	6%	<0.0001
**HbA1c Measurement Year**					
2003	6%	8%	5%	4%	
2006	27%	26%	28%	27%	
2008	27%	28%	26%	26%	
2010	14%	14%	14%	14%	
2012	13%	12%	14%	14%	
2014	5%	5%	5%	5%	
2016	8%	7%	8%	10%	
**Median HbA1c (IQR)**	5.7% (5.3%-6.2%)	5.8% (5.5%-6.4%)	5.7% (5.2%-6.2%)	5.6% (5.2%-6.0%)	
**Proportion Pre-Diabetic**	30%	34%	30%	26%	<0.0001

**(HbA1c between 5.71% - 6.49%)**					
**Proportion Diabetic (HbA1c ≥ 6.5%)**	17%	23%	16%	12%	<0.0001
**Medication (Yes)**	19%	28%	19%	13%	<0.0001

Note: Reported average mother and father's education includes participants with missing values that were replaced with the sample mean (mean of 10 years for both mother's and father's education). Chi-square tests are used to compare significant differences in categorical variables by education level.

Abbreviations: IQR = interquartile range

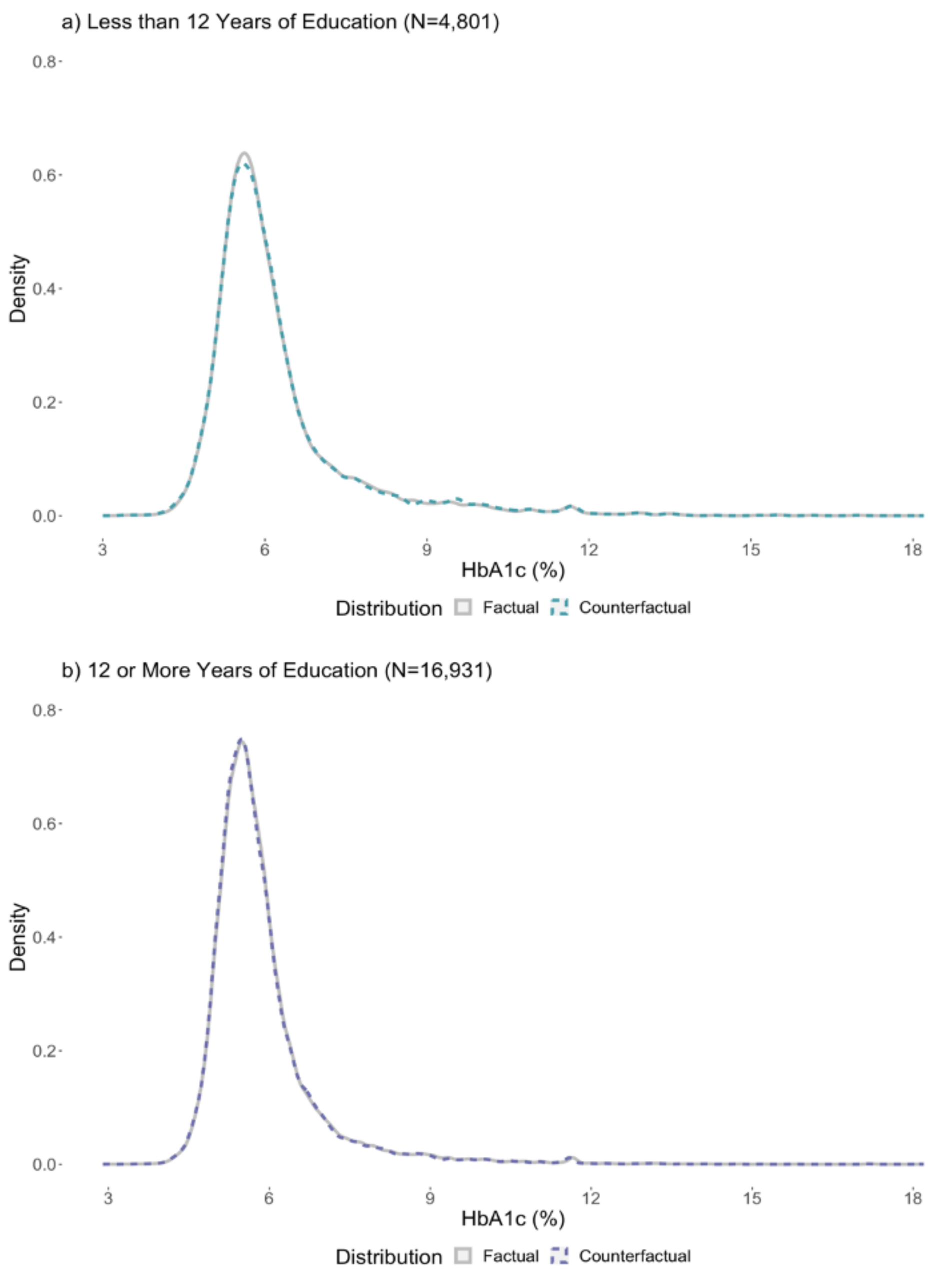

eFigure 1. Counterfactual HbA1c distributions based on unconditional quantile regression estimates by dichotomized education

Note: Counterfactual HbA1c distributions were created using quantile estimates from UQR models. Counterfactual distributions were created to help visualize the reshaping of the HbA1c distribution for a one-year increase in the average educational attainment in the sample.

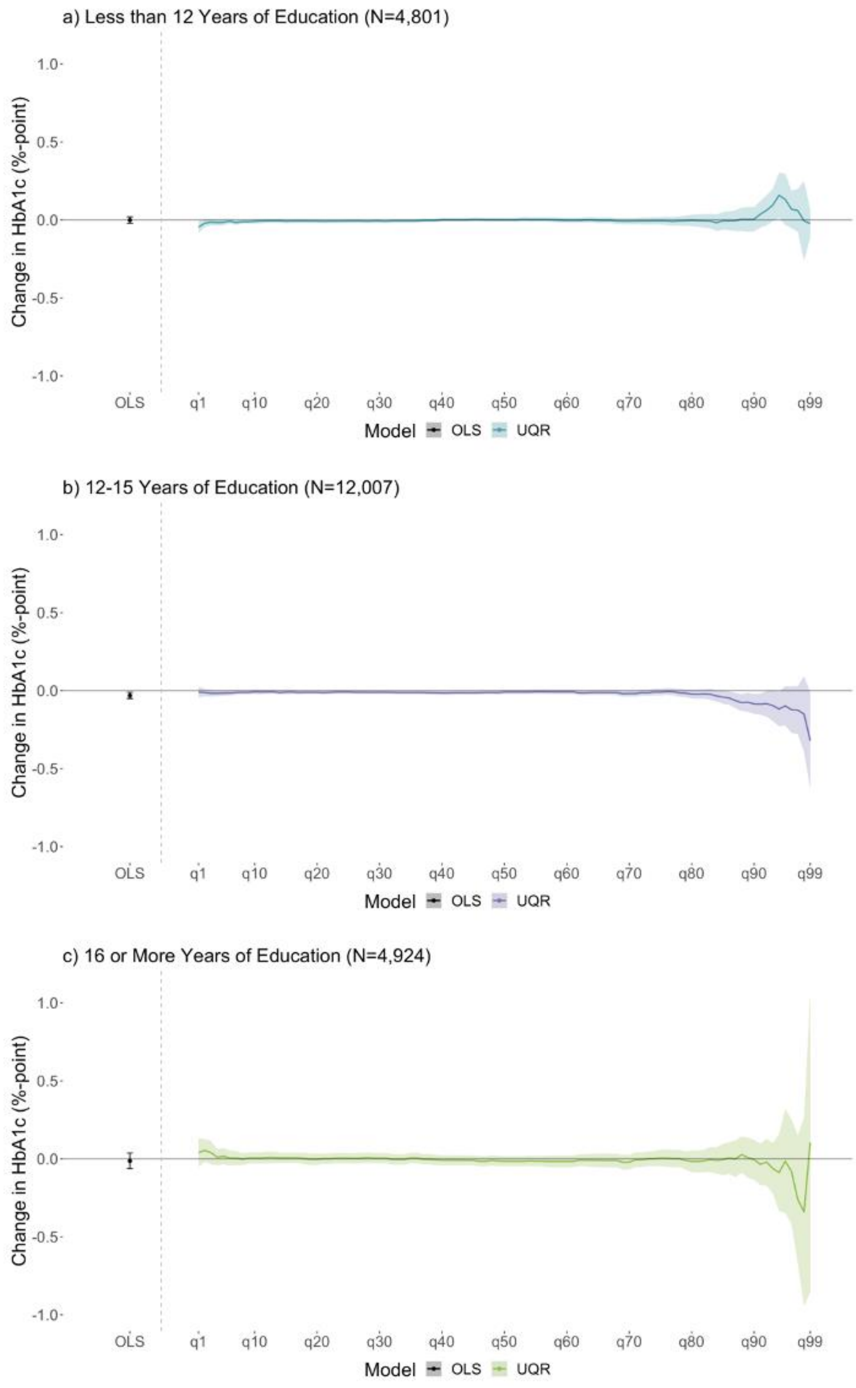

eFigure 2. Comparing results from linear regressions estimated using ordinary least squares with unconditional quantile regressions by 3-level education

Notes: OLS stands for Ordinary Least Squares. q10 = 10th quantile, q20 = 20th quantile, and so forth. The point to the left of the vertical dashed gray line in each panel represents the point estimate from OLS with 95% confidence

intervals. The solid line to the right of the vertical dashed gray line in each panel represents point estimates from unconditional quantile regressions and shaded areas represent the 95% confidence intervals fit at each unit quantile between the 1st-99th quantiles of the HbA1c distribution. All models were adjusted for covariates and 95% confidence intervals were estimated using bootstrapping (500 resamples).

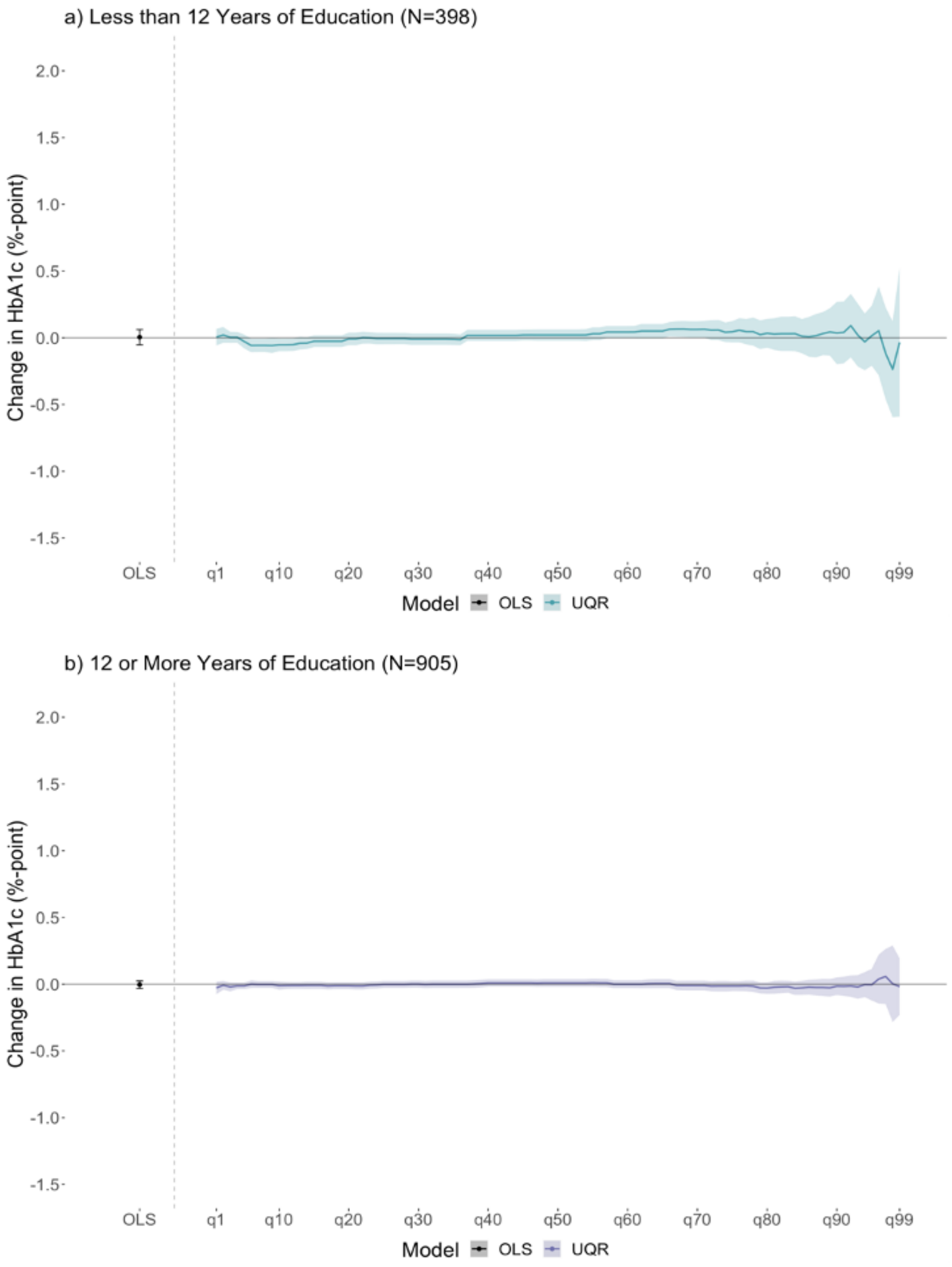

eFigure 3. Comparing AHEAD Cohort results from linear regressions estimated using ordinary least squares with unconditional quantile regressions by dichotomized education

Notes: AHEAD stands for The Study of Assets and Health Dynamics Among the Oldest Old (birth year range: 1900-1923). OLS stands for Ordinary Least Squares. q10 = 10th quantile, q20 = 20th quantile, and so forth. The point to the left of the vertical dashed gray line in each panel represents the point estimate from OLS with 95% confidence intervals. The solid line to the right of the vertical dashed gray line in each panel represents point

estimates from unconditional quantile regressions and shaded areas represent the 95% confidence intervals fit at each unit quantile between the 1st-99th quantiles of the HbA1c distribution. All models were adjusted for covariates and 95% confidence intervals were estimated using bootstrapping (500 resamples). For participants with less than 12 years of education, a one-year increase in mean education was associated with lower HbA1c at higher quantiles of the unconditional distribution. A one-year increase in mean education was not associated with HbA1c for participants with 12 or more years of education. Differences in results by birth cohort compared to main results are most likely due to the smaller number of participants within each education strata: there is high variation in the participants that fall in the edges of the HbA1c distribution creating unstable estimates, exemplified by the wider confidence intervals at higher quantiles of the distribution.

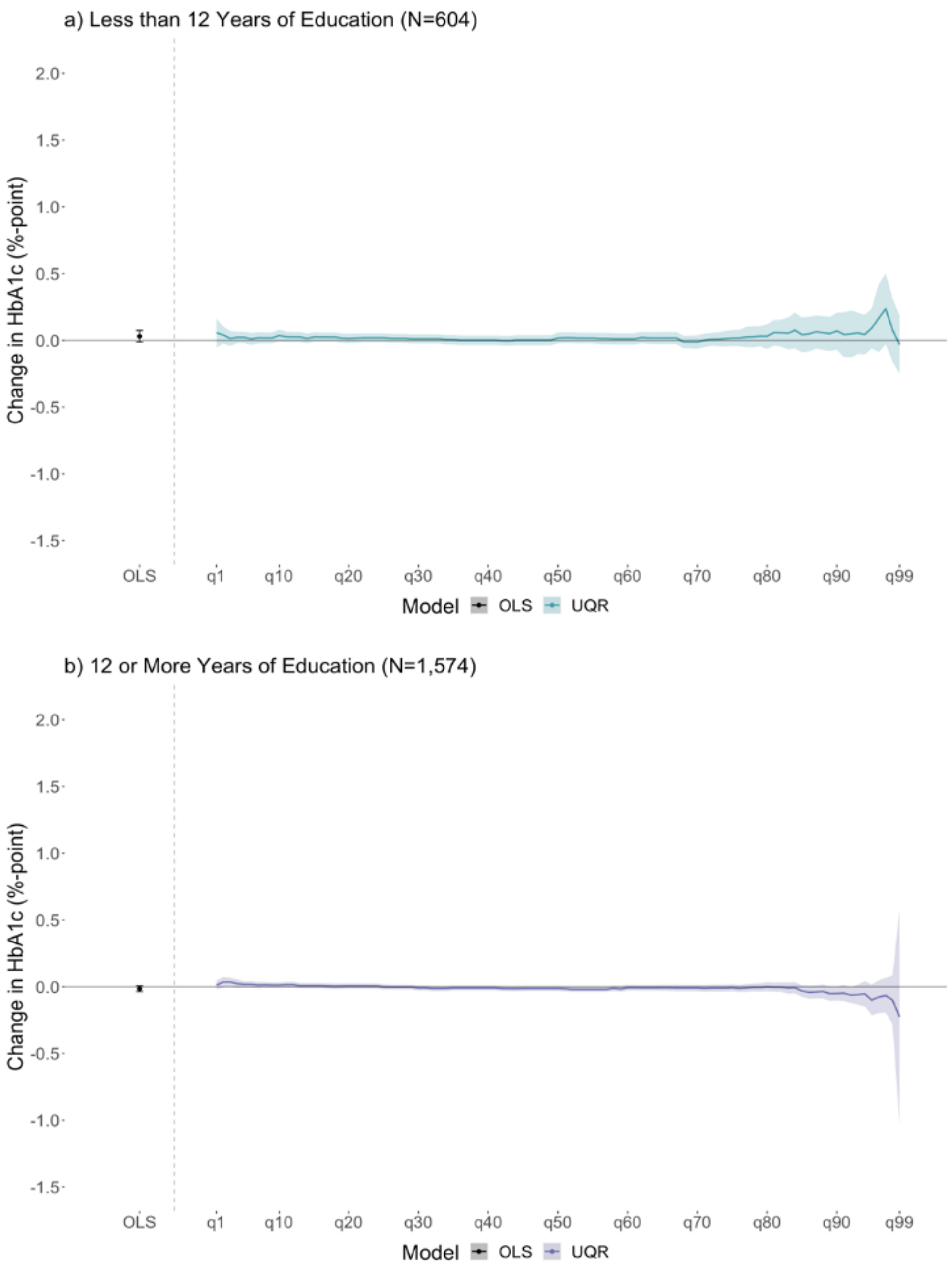

eFigure 4. Comparing CODA Cohort results from linear regressions estimated using ordinary least squares with unconditional quantile regressions by dichotomized education

Notes: CODA stands for Children of Depression (birth year range: 1924-1930). OLS stands for Ordinary Least Squares. q10 = 10th quantile, q20 = 20th quantile, and so forth. The point to the left of the vertical dashed gray line in each panel represents the point estimate from OLS with 95% confidence intervals. The solid line to the right of the vertical dashed gray line in each panel represents point estimates from unconditional quantile regressions and

shaded areas represent the 95% confidence intervals fit at each unit quantile between the 1st-99th quantiles of the HbA1c distribution. All models were adjusted for covariates and 95% confidence intervals were estimated using bootstrapping (500 resamples). For participants with less than 12 years of education, a one-year increase in mean education was associated with higher HbA1c at higher quantiles of the unconditional distribution. For participants with 12 or more years of education, a one-year increase in mean education was associated with lower HbA1c at higher quantiles of the unconditional distribution. Differences in results by birth cohort compared to main results are most likely due to the smaller number of participants within each education strata: there is high variation in the participants that fall in the edges of the HbA1c distribution creating unstable estimates, exemplified by the wider confidence intervals at higher quantiles of the distribution.

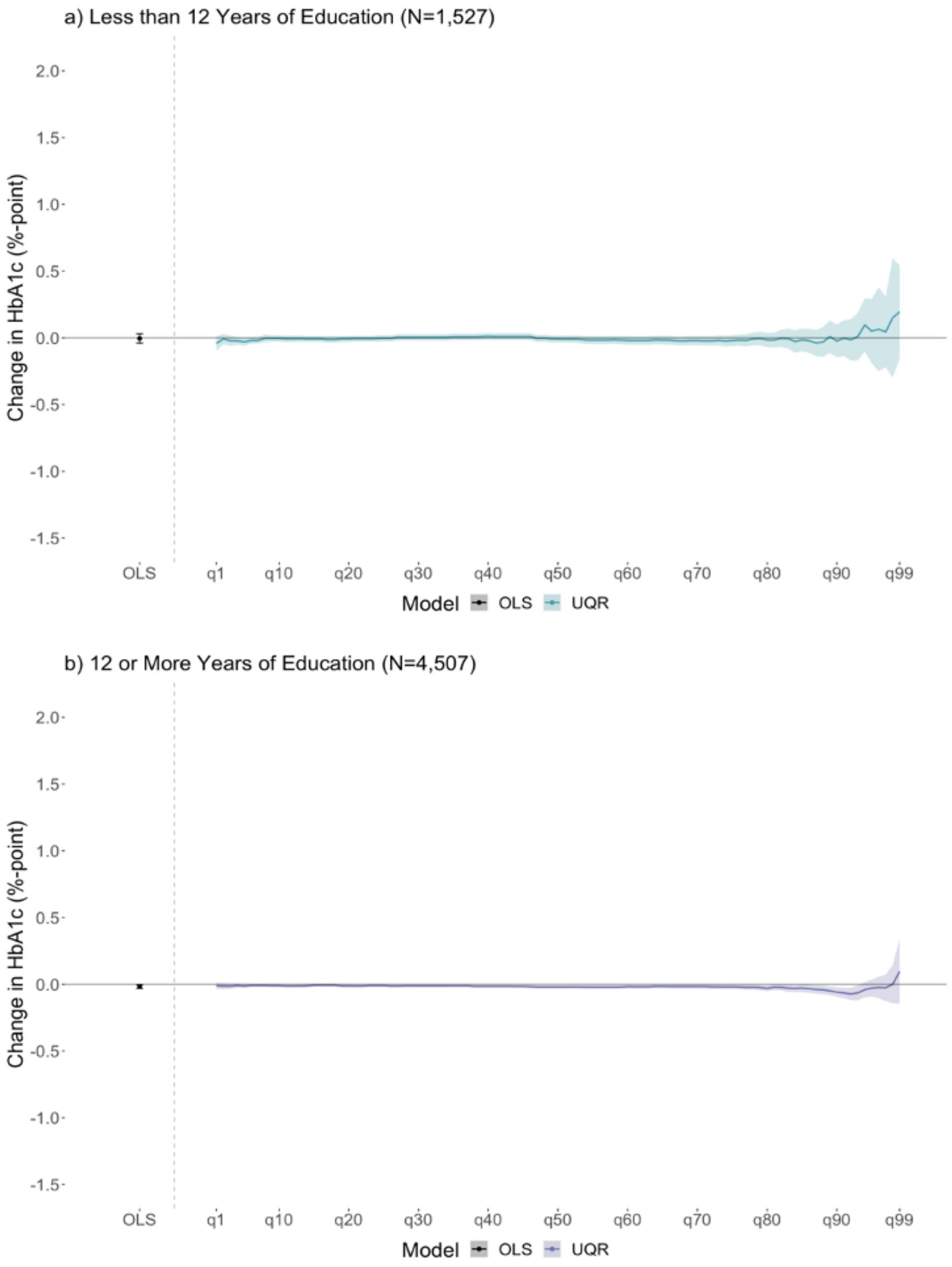

eFigure 5. Comparing HRS Cohort results from linear regressions estimated using ordinary least squares with unconditional quantile regressions by dichotomized education

Notes: HRS stands for Initial HRS cohort (birth year range: 1931-1941). OLS stands for Ordinary Least Squares. q10 = 10th quantile, q20 = 20th quantile, and so forth. The point to the left of the vertical dashed gray line in each panel represents the point estimate from OLS with 95% confidence intervals. The solid line to the right of the vertical dashed gray line in each panel represents point estimates from unconditional quantile regressions and shaded

areas represent the 95% confidence intervals fit at each unit quantile between the 1st-99th quantiles of the HbA1c distribution. All models were adjusted for covariates and 95% confidence intervals were estimated using bootstrapping (500 resamples). For participants with less than 12 years of education, a one-year increase in mean education was associated with higher HbA1c at higher quantiles of the unconditional distribution. For participants with 12 or more years of education, a one-year increase in mean education was associated with lower HbA1c across most quantiles but was associated with higher HbA1c at the highest quantile (99th quantile). Differences in results by birth cohort compared to main results are most likely due to the smaller number of participants within each education strata: there is high variation in the participants that fall in the edges of the HbA1c distribution creating unstable estimates, exemplified by the wider confidence intervals at higher quantiles of the distribution.

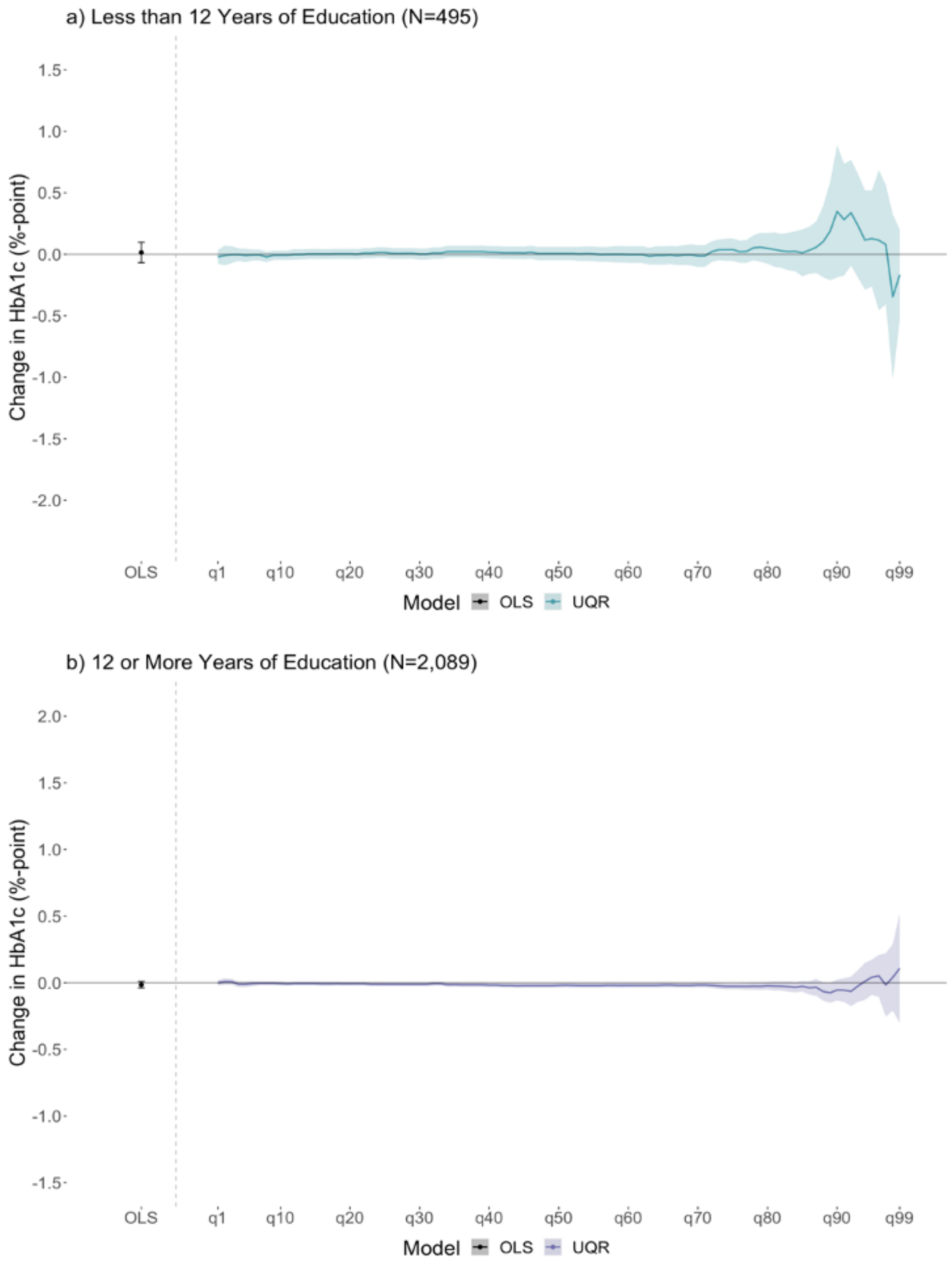

eFigure 6. Comparing WB Cohort results from linear regressions estimated using ordinary least squares with unconditional quantile regressions by dichotomized education

Notes: WB stands for War Baby cohort (birth year range: 1942-1947). Note that for the 16 or more years of schooling group, the 95% confidence intervals for the 99th quantile have been cut off to maintain the same y-axis scale for all groups (true 95% CI range: -427, 439). OLS stands for Ordinary Least Squares. q10 = 10th quantile, q20 = 20th quantile, and so forth. The point to the left of the vertical dashed gray line in each panel represents the

point estimate from OLS with 95% confidence intervals. The solid line to the right of the vertical dashed gray line in each panel represents point estimates from unconditional quantile regressions and shaded areas represent the 95% confidence intervals fit at each unit quantile between the 1st-99th quantiles of the HbA1c distribution. All models were adjusted for covariates and 95% confidence intervals were estimated using bootstrapping (500 resamples). For participants with less than 12 years of education, a one-year increase in mean education was associated with higher HbA1c at the highest quantiles of the unconditional distribution (72-96th quantiles) but is associated with lower HbA1c at the highest quantiles (98-99th quantiles). For participants with 12 or more years of education, a one-year increase in mean education was associated with lower HbA1c across most quantiles but was associated with higher HbA1c at the highest quantiles of the unconditional distribution (94-96th and 98-99th quantiles). Differences in results by birth cohort compared to main results are most likely due to the smaller number of participants within each education strata: there is high variation in the participants that fall in the edges of the HbA1c distribution creating unstable estimates, exemplified by the wider confidence intervals at higher quantiles of the distribution.

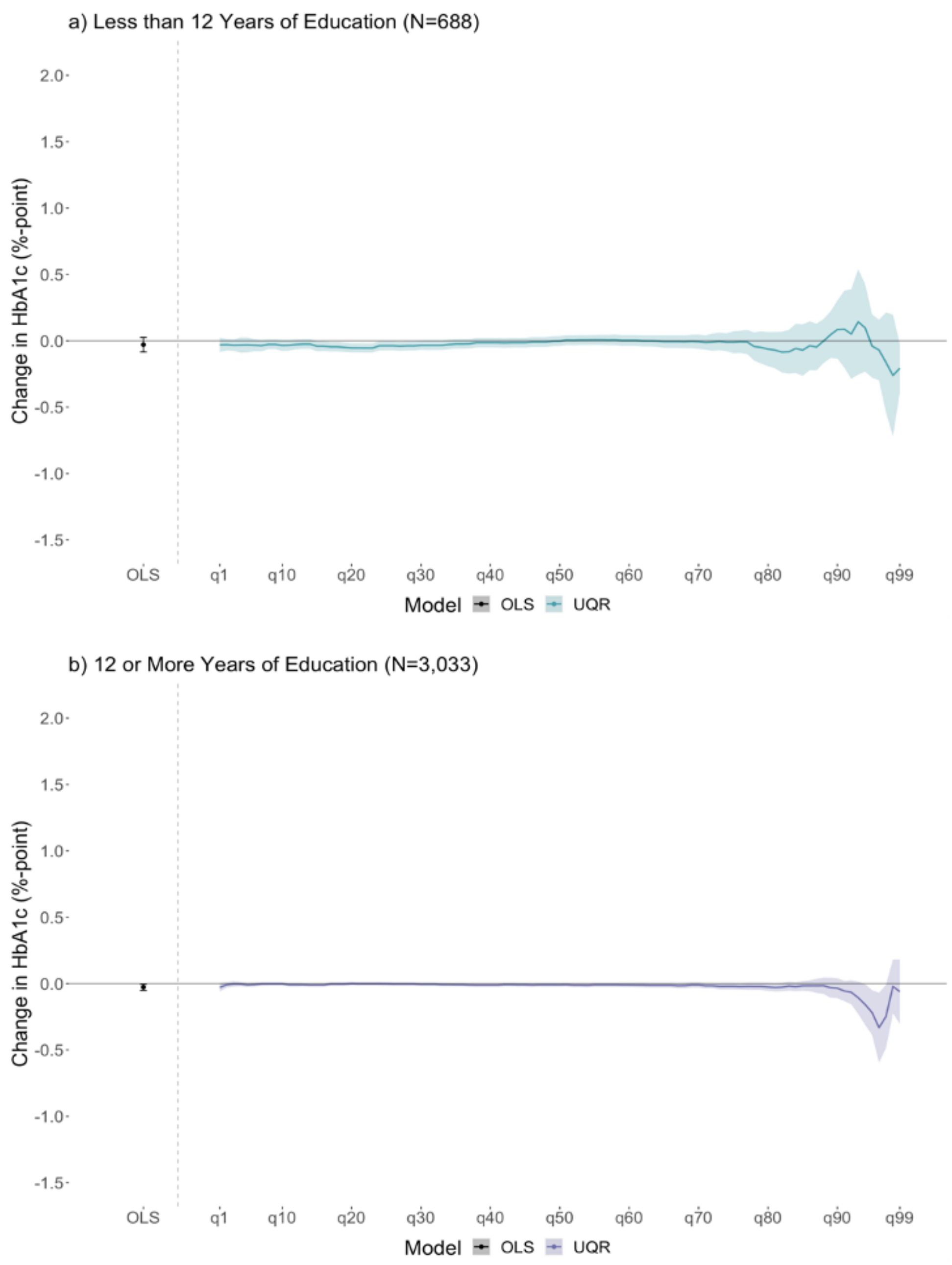

eFigure 7. Comparing EBB Cohort results from linear regressions estimated using ordinary least squares with unconditional quantile regressions by dichotomized education

Notes: EBB stands for Early Baby Boomer cohort (birth year range: 1948-1953). OLS stands for Ordinary Least Squares. q10 = 10th quantile, q20 = 20th quantile, and so forth. The point to the left of the vertical dashed gray line in each panel represents the point estimate from OLS with 95% confidence intervals. The solid line to the right of the vertical dashed gray line in each panel represents point estimates from unconditional quantile regressions and

shaded areas represent the 95% confidence intervals fit at each unit quantile between the 1st-99th quantiles of the HbA1c distribution. All models were adjusted for covariates and 95% confidence intervals were estimated using bootstrapping (500 resamples). For participants with less than 12 years of education, a one-year increase in mean education was associated with lower HbA1c except for some quantiles (51-61st and 90-94th quantiles). For participants with more than 12 years of education, a one-year increase in mean education was associated with lower HbA1c and magnitudes were larger at higher quantiles of the unconditional distribution. Differences in results by birth cohort compared to main results are most likely due to the smaller number of participants within each education strata: there is high variation in the participants that fall in the edges of the HbA1c distribution creating unstable estimates, exemplified by the wider confidence intervals at higher quantiles of the distribution.

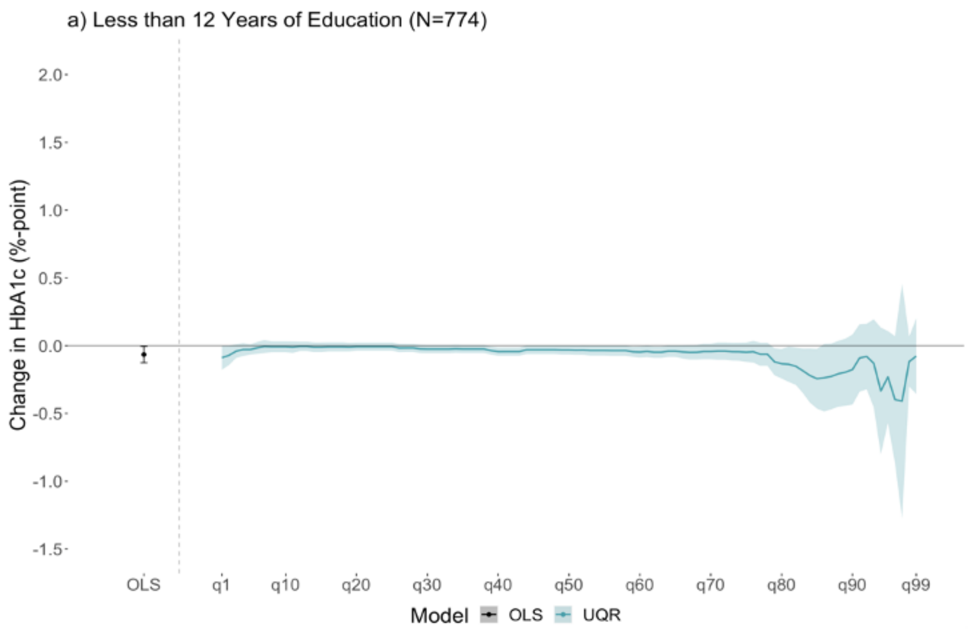

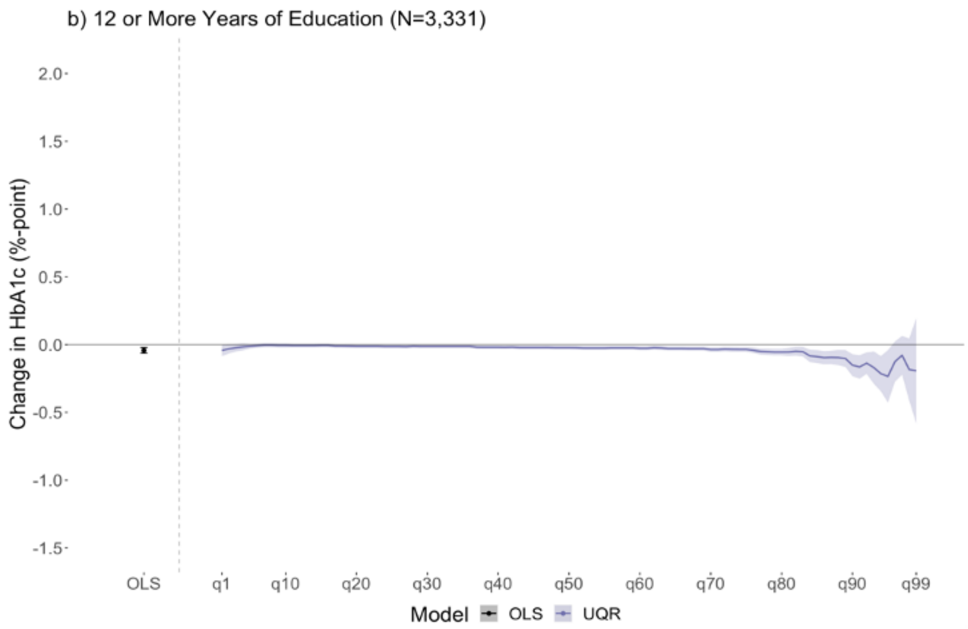

eFigure 8. Comparing MBB Cohort results from linear regressions estimated using ordinary least squares with unconditional quantile regressions by dichotomized education

Notes: MBB stands for Mid Baby Boomer cohort (birth year range: 1954-1959). OLS stands for Ordinary Least Squares. q10 = 10th quantile, q20 = 20th quantile, and so forth. The point to the left of the vertical dashed gray line in each panel represents the point estimate from OLS with 95% confidence intervals. The solid line to the right of the vertical dashed gray line in each panel represents point estimates from unconditional quantile regressions and

shaded areas represent the 95% confidence intervals fit at each unit quantile between the 1st-99th quantiles of the HbA1c distribution. All models were adjusted for covariates and 95% confidence intervals were estimated using bootstrapping (500 resamples). For participants with less than 12 and 12 or more years of education, a one-year increase in mean education was associated with lower HbA1c across all quantiles, and the magnitude was larger at higher quantiles of the distribution. Differences in results by birth cohort compared to main results are most likely due to the smaller number of participants within each education strata: there is high variation in the participants that fall in the edges of the HbA1c distribution creating unstable estimates, exemplified by the wider confidence intervals at higher quantiles of the distribution.

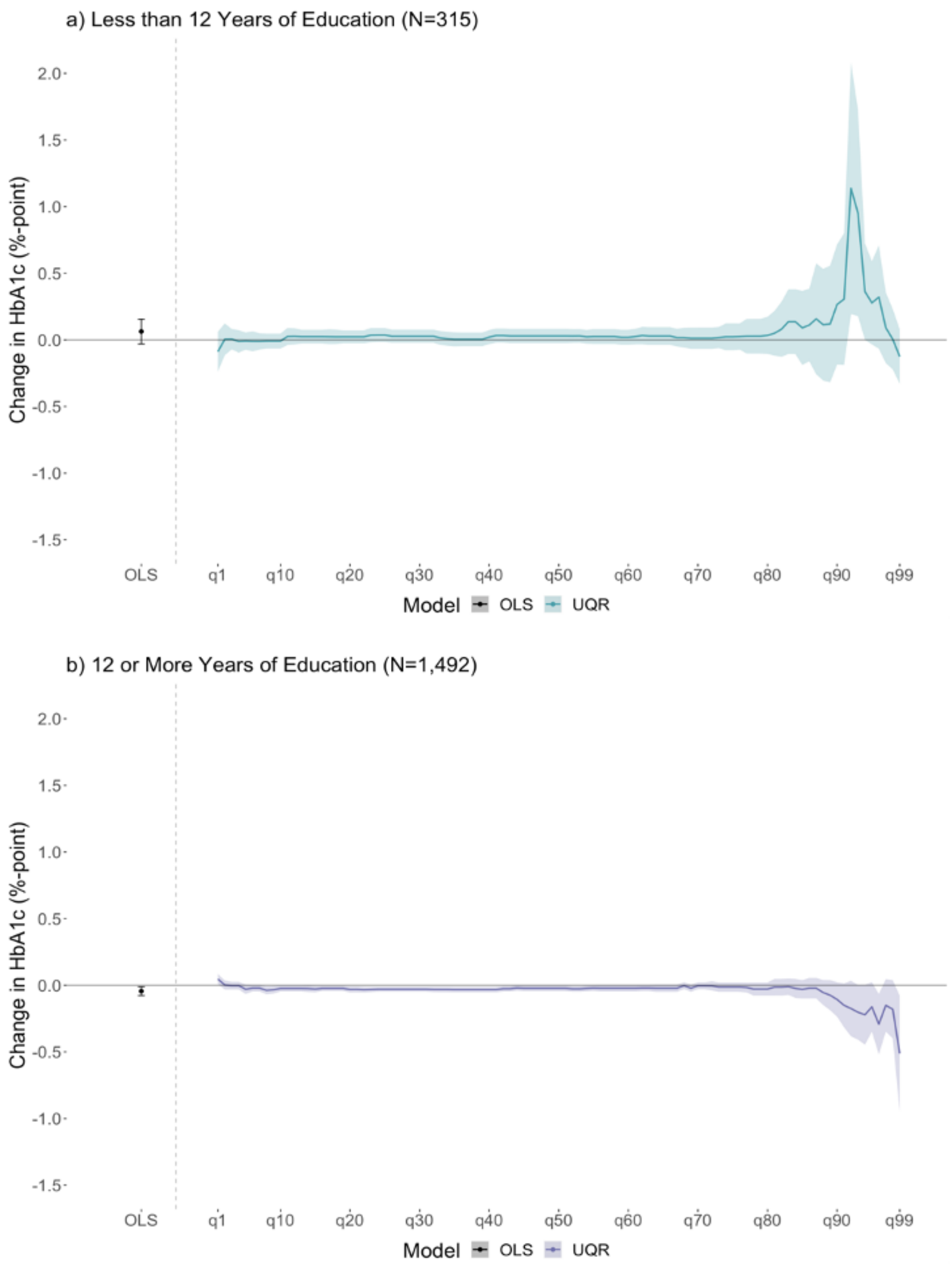

eFigure 9. Comparing LBB Cohort results from linear regressions estimated using ordinary least squares with unconditional quantile regressions by dichotomized education

Notes: LBB stands for Late Baby Boomer cohort (birth year range: 1960-1965). OLS stands for Ordinary Least Squares. q10 = 10th quantile, q20 = 20th quantile, and so forth. The point to the left of the vertical dashed gray line in each panel represents the point estimate from OLS with 95% confidence intervals. The solid line to the right of the vertical dashed gray line in each panel represents point estimates from unconditional quantile regressions and

shaded areas represent the 95% confidence intervals fit at each unit quantile between the 1st-99th quantiles of the HbA1c distribution. All models were adjusted for covariates and 95% confidence intervals were estimated using bootstrapping (500 resamples). For participants with less than 12 years of education, a one-year increase in mean education was associated with higher HbA1c at higher quantiles of the unconditional distribution. For participants with 12 or more years of education, a one-year increase in mean education was associated with lower HbA1c at higher quantiles of the unconditional distribution. Differences in results by birth cohort compared to main results are most likely due to the smaller number of participants within each education strata: there is high variation in the participants that fall in the edges of the HbA1c distribution creating unstable estimates, exemplified by the wider confidence intervals at higher quantiles of the distribution.

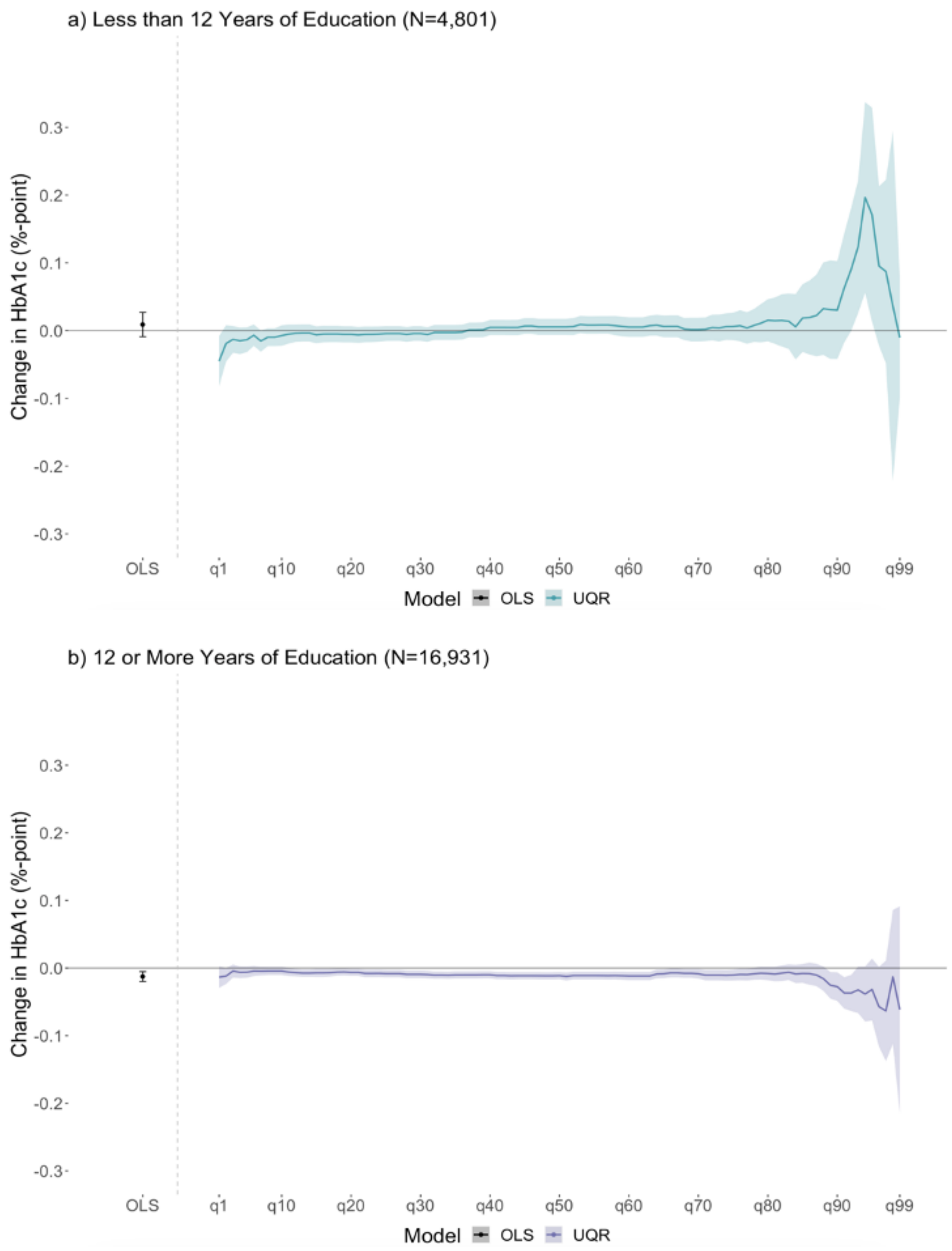

eFigure 10. Comparing results from linear regressions estimated using ordinary least squares with unconditional quantile regressions by dichotomized education when models are additionally adjusted for medication

Notes: OLS stands for Ordinary Least Squares. q10 = 10th quantile, q20 = 20th quantile, and so forth. The point to the left of the vertical dashed gray line in each panel represents the point estimate from OLS with 95% confidence intervals. The solid line to the right of the vertical dashed gray line in each panel represents point estimates from

unconditional quantile regressions and shaded areas represent the 95% confidence intervals fit at each unit quantile between the 1st-99th quantiles of the HbA1c distribution. All models were adjusted for covariates and 95% confidence intervals were estimated using bootstrapping (500 resamples).
